# Neurosyphilis Presenting with Anxiety: A Case Report

**DOI:** 10.7759/cureus.3020

**Published:** 2018-07-22

**Authors:** Ashley N Rubin, Eduardo D Espiridion, Nhu-Hac Truong, Daniel H Lofgren

**Affiliations:** 1 Family Medicine, West Virginia School of Osteopathic Medicine, Lewisburg, USA; 2 Psychiatry, Frederick Memorial Hospital, Frederick, USA; 3 Family Medicine, West Virginia School of Osteopathic Medicine, Silver Spring, USA; 4 Surgery Student, West Virginia School of Osteopathic Medicine, Lewisburg , USA

**Keywords:** neurosyphilis, argyll-robertson pupils, altered mental status, public health, geriatrics, syphilis, anxiety

## Abstract

The number of cases of late and late latent syphilis in the United States is on the rise. This diagnosis is often forgotten when an elderly patient is being worked up for altered mental status. Rarely does a 70-year-old male with neurosyphilis present simply with anxiety. Due to the decreased severity of the presentation, this patient was sent home from the emergency department multiple times until the anxiety progressed to psychosis. He was finally admitted with delirium, suicidal ideation, and paranoia. A routine *Treponema pallidum* antibody test returned positive and a further workup of confirmatory lab work, a thorough neurological exam, and magnetic resonance imaging (MRI) revealed a chronic syphilis infection. This case study explores signs in the history and physical examination that should quickly prompt a provider to consider neurosyphilis in their differential. This patient presented with Argyll-Robertson pupils and significant risk factors. The goal of this discussion is to bring awareness to this infrequent presentation and share simple examination techniques that could have been used to diagnose and treat this patient’s symptoms more promptly. In doing so, the hope is to raise awareness for the diagnosis of neurosyphilis, especially in the elderly patient presenting with psychiatric symptoms.

## Introduction

Neurosyphilis is the most feared and severe manifestation of syphilis. Neurosyphilis occurs when *Treponema pallidum* infiltrates the central nervous system of a patient. Although the prevalence of syphilis in the United States dropped dramatically after the wide-spread use of antibiotics, the rates began to slowly increase yearly since 1993. Specifically, the number of cases of late and late latent syphilis increased by 17.2% yearly [1]. The risk factors to contract syphilis include male gender, the use of cocaine, the increased use of methamphetamine, and viagra [2-3]. Today, neurosyphilis is most commonly seen in the human immunodeficiency virus (HIV) population [2]. The risk factors of neurosyphilis in human immunodeficiency (HIV)-negative patients include Caucasian males and age over 45 years [4].

To diagnose neurosyphilis, a patient must first be confirmed to be infected with *Treponema pallidum*. To begin testing for syphilis, simple serum tests can be performed, including venereal disease research laboratory (VDRL) testing and rapid plasma reagin (RPR) testing. It can also be detected with serum treponemal tests, which include the fluorescent treponemal antibody absorption (FTA-ABS) test, *Treponema pallidum* particle agglutination assay (TPPA), and syphilis enzyme immunoassays (EIAs). Regrettably, nontreponemal tests, such as RPR and VDRL, can be non-reactive in neurosyphilis [5]. In these cases, serum treponemal tests, such as FTA-ABS, TPPA, or EIA, should always be performed because these tests remain reactive throughout life, regardless of prior treatment [4]. Positive serum treponemal tests confirm that the patient has been infected with syphilis during his/her lifetime [4]. In patients with a known syphilis infection who present with neurologic, ophthalmic, or tertiary syphilis symptoms, the United States Centers for Disease Control and Prevention (CDC) recommends a lumbar puncture with a cerebrospinal fluid (CSF) examination. The importance of identifying CSF abnormalities is not necessarily for the identification of infection, it is rather to help assess the efficacy of treatment [4,6].

Neurosyphilis is one of the most complicated manifestations to diagnose without a known history of syphilis infection due to its vast possible presentations. The most common presentations of neurosyphilis include tabes dorsalis, general paresis, and meningovascular neurosyphilis. Meningovascular neurosyphilis will cause stroke-like symptoms due to its endarteritis with perivascular brain inflammation [4,6]. It can occur anytime between the first few months to several years after the initial infection and will cause stroke-like symptoms in commonly young adult populations [4,6]. General paresis usually occurs anytime from three to 30 years after infection, peaking 25 years after initial exposure [6]. General paresis symptoms include personality changes, hyperreflexia, Argyll-Robertson pupils, sensory changes, such as delusions and hallucinations, and changes in speech [6-7]. These symptoms are caused by progressive frontotemporal meningoencephalitis with diffuse cortical atrophy [7]. Tabes dorsalis can occur anytime from five to 50 years after infection and typically surfaces around 20 years after infection [8]. It is caused by a slowly progressing demyelination of the spinal cords’ posterior roots and columns [8]. Symptoms of tabes dorsalis include excruciating pain and sensory ataxia. Argyll-Robertson pupils are very specific to neurosyphilis and are most commonly seen with the tabes dorsalis presentation [9].

## Case presentation

A 70-year-old African American male was seen in the emergency department for acute anxiety and paranoia. He reported that his son gave him melatonin to help him sleep, but he felt the medication was poisonous. He also reported that he was struggling with the death of his partner of 40 years and was feeling anxious. The patient was prescribed risperidone and lorazepam and was discharged shortly thereafter. Twenty-four hours later, he was seen again in the emergency department for worsening anxiety, psychosis, suicidal ideation, and command hallucinations. The patient's son reported that his father “had not slept in weeks.” The son reported progressive agitation, paranoia, and bizarre behavior. Due to the worsening psychosis, he was admitted to the medical floor for further work-up. He reported feeling as though people were watching him and that someone was going to harm him. When questioned about his reported suicidal ideation, he blamed that thought on his post-traumatic stress disorder; but would not elaborate on the event. The patient was given a one-to-one sitter due to his suicidal ideation.

This patient lives with his family of seven children. He has a history of prolonged incarceration. His past medical history is significant for hypertension and negative for seizure disorders. The patient had never been hospitalized for psychiatric issues prior to this visit. He denied past suicidal behavior and any history of physically or sexually aggressive behavior. The patient reported a history of excessive alcohol abuse for more than a year following the death of his partner. He reported that he stopped drinking “cold turkey” five months prior to this visit. The patient's family history is significant for a son with an anxiety disorder. He denied a history of physical or sexual abuse in the past.

The patient's mental status exam was significant for an anxious affect with referential and paranoid ideations. He denied any thought broadcasting, insertion, or withdrawal. He had some paranoid and persecutory delusions. Insight, judgment, and impulse control were poor.

The patient's initial physical exam revealed an inability to ambulate and overall weakness. Further neurological examination revealed mental disorientation with bilateral muscle wasting, sensory deficit, and hyporeflexive ankles. Additionally, the patient exhibited stance and gait abnormalities. A later eye exam revealed bilateral light-near dissociation with accommodation but no reaction to light. Otherwise, all other neurological components were intact and within normal limits.

Initial routine testing was positive for the *Treponema pallidum* antibody, suggesting prior infection, but the distinction between treated and untreated syphilis cannot be made as the Treponemal-specific immunoglobulin G (IgG) may remain elevated throughout life. An RPR was then ordered to help distinguish between acute or chronic infection and a *Treponema pallidum* particle agglutination test was ordered to distinguish between syphilis infection and a false positive screening test. The RPR returned nonreactive but the *Treponema pallidum* particle agglutination returned reactive, indicating a prior infection of *Treponema pallidum*. The initial differential diagnosis workup included testing to exclude thiamine, folate and B12 deficiency, insomnia, hypocalcemia, hypothyroidism/hyperthyroidism, HIV encephalopathy, dementia, stroke, drug or alcohol intoxication, and normal pressure hydrocephalus.

Further labs showed white blood cell count, sodium, potassium, calcium, anion gap, creatinine, glomerular filtration rate, glucose, mean corpuscular volume, and thyroid stimulating hormone within normal limits. Urinalysis, toxicology screening, alcohol levels, and HIV antibody were also negative. Imaging included a head computed tomography (CT), which showed no abnormalities, a brain magnetic resonance imaging (MRI) that showed cortical atrophy, and an electroencephalogram within normal limits.

## Discussion

The topic of neurosyphilis, particularly within the geriatrics population, has not been examined in major published literature in recent years. This is largely due in part to the advent of widespread antibiotic usage which ultimately prevents the progression of syphilis [10]. As such, neurosyphilis cases are now mainly associated with HIV patients and men who have sex with men [11]. To make matters more complicated, of the 25 to 35 percent of syphilis patients who develop neurosyphilis, one third are asymptomatic. Another third of those patients develop tabes dorsalis and, of those people, only half have concomitant Argyll-Robertson pupils [12]. General paresis tends to become obvious 10 to 25 years after the initial infection and has been shown to be missed in about 36% of patients presenting with “dementia, personality change, abnormal behavior, and emotional problems,” thereby preventing proper treatment for one to 24 months [13]. Therefore, the current national census of neurosyphilis remains statistically unclear due to many unreported cases [14].

**Figure 1:**
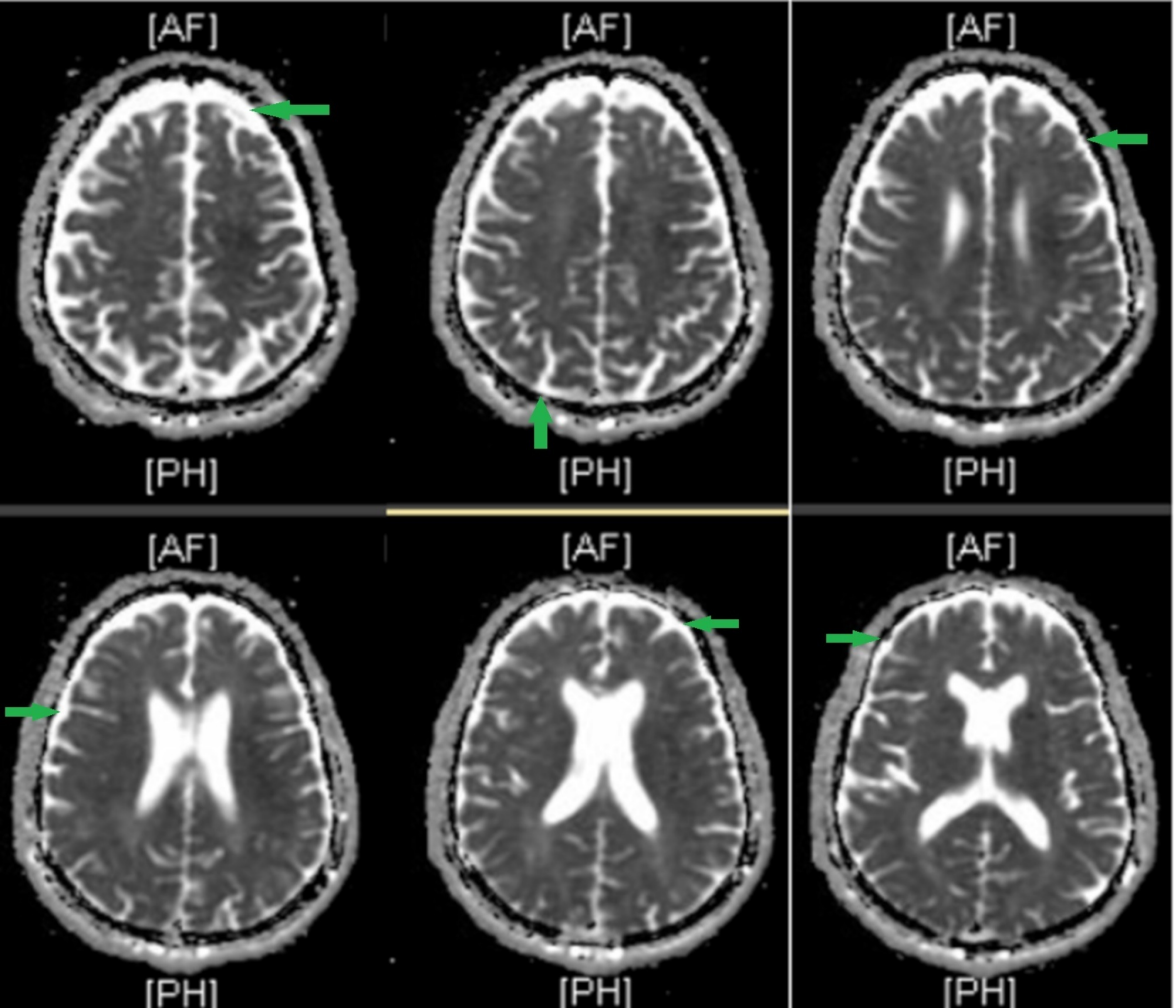
T2-weighted brain MRI of a 70-year-old male with neurosyphilis showing diffuse cortical atrophy. Green arrows indicate areas of cortical atrophy.

What makes this case so striking is that not only is neurosyphilis a rarity to encounter, but this patient also displayed neurological symptoms that diverge from the usual disease process presentation. He presented with acute anxiety, delirium with paranoia, and command hallucinations that were, at the time, attributed to possible Wernicke’s encephalopathy secondary to prolonged alcohol abuse. It was only after a routine positive RPR test that a TPPA test was ordered and ultimately returned positive as well. A complete neurological exam was subsequently performed, resulting in signs significant of tabes dorsalis: bilateral muscle wasting, sensory deficits, and hyporeflexive ankles. Moreover, MRI studies revealed cortical thinning, substantiating the presence of general paresis (Figure 1). In addition to this, the pupils were accommodative but non-reactive to light. The presence of Argyll-Robertson pupils in the setting of general paresis is not unusual, but the ocular finding is usually more indicative of tabes dorsalis development [15].

Looking retrospectively at his risk factors for progressive chronic infection, his older age and social history appear to play a role, as well as his history of alcohol abuse and psychotic agitation after years of incarceration. His race also served as a risk factor, as African Americans are 4.7 times more likely to contract the disease [1].

## Conclusions

This case highlights the importance of considering neurosyphilis on the differential list, especially in the elderly patient population. Due to the patient's longstanding history of substance abuse and imprisonment, he is a prime example of a chronic *Treponema pallidum* infection that manifested under the disguise of an acute psychiatric and/or alcohol withdrawal process. The difficulty that the public health realm faces with this issue is that many of the clinical neurosyphilis symptoms in elderly patients can imitate more common neurologic or psychiatric diseases, such as dementia, psychiatric disorders, delirium, and stroke. As such, it is important to include a syphilis workup as part of the routine management of an elderly patient presenting with psychiatric and/or neurological symptoms.

## References

[1] Centers for Disease Control and Prevention (2018). 2016 Sexually Transmitted Diseases Surveillance. http://www.cdc.gov/std/stats16/syphilis.htm.

[2] Wong W, Chaw JK, Kent CK, Klausner JD (2005). Risk factors for early syphilis among gay and bisexual men seen in an STD clinic: San Francisco, 2002-2003. J Sex Transm Dis.

[3] Golden MR, Marra CM, Holmes KK (2003). Update on syphilis: resurgence of an old problem. JAMA.

[4] Read PJ, Donovan B (2012). Clinical aspects of adult syphilis. Internal Med J.

[5] Van der Sluis JJ (1992). Laboratory techniques in the diagnosis of syphilis: a review. Genitourin Med.

[6] Shi M, Peng R, Gao Z (2016). Risk profiles of neurosyphilis in HIV‐negative patients with primary, secondary and latent syphilis: implications for clinical intervention. J Eur Acad Dermatol Venereol.

[7] Yanhua W, Haishan S, Le H (2018). Clinical and neuropsychological characteristics of general paresis misdiagnosed as primary psychiatric disease. BMC Psychiatry.

[8] Pandey S (2011). Magnetic resonance imaging of the spinal cord in a man with tabes dorsalis. J Spinal Cord Med.

[9] Pearce JMS (2004). The Argyll Robertson pupil. J Neurol Neurosurg Psychiatry.

[10] Workowski KA, Bolan GA (2018). Centers for Disease Control and Prevention. Sexually transmitted diseases treatment guidelines. Erratum in: MMWR Recomm Rep.

[11] Gray RT, Hoare A, Prestage GP, Donovan B, Kaldor JM, Wilson DP (2010). Frequent testing of highly sexually active gay men is required to control syphilis. J Sex Transm Dis.

[12] Merritt HH (1939). Neurosyphilis and its treatment. N Engl J Med.

[13] Stokes JH, Beerman H (1946). Modern clinical syphilology diagnosis, treatment, case study. Sex Transm Infect.

[14] Dong Z, Daoyou Z, Zhongyan Z (2011). The clinical presentation and imaging manifestation of psychosis and dementia in general paresis: a retrospective study of 116 cases. J Neuropsychiatry Clin Neurosci.

[15] Zetola NM, Engelman J, Jensen TP, Klausner JD (2007). Syphilis in the United States: an update for clinicians with an emphasis on HIV coinfection. Mayo Clin Proc.

